# Calciprotein Particles in CKD: When Good Molecules Go Bad

**DOI:** 10.34067/KID.0000000782

**Published:** 2025-04-24

**Authors:** Lorenza Magagnoli, Mario Cozzolino

**Affiliations:** Department of Health Sciences, University of Milan, Milan, Italy and Renal Division, ASST Santi Paolo e Carlo, Milan, Italy

**Keywords:** CKD, mineral metabolism, mortality, progression, biomarkers

Every nephrologist is well aware of the disturbances in mineral metabolism that accompany CKD, contributing to a high risk of bone disease and extraskeletal calcifications, associated with higher morbidity and mortality.^1^ Serum calcium, phosphate, parathyroid hormone (PTH), and vitamin D levels are closely monitored and routinely addressed in clinical practice. Yet, beyond these familiar markers lies a lesser-known, but potentially crucial, player: calciprotein particles (CPPs). Despite growing evidence linking CPPs to vascular calcification, inflammation, and cardiovascular risk, many nephrologists remain unfamiliar with their biology, clinical significance, and potential utility.^2^ Could these microscopic structures offer new insights into CKD management?

CPPs are circulating blood-borne nanoparticles composed of calcium, phosphate, and serum proteins such as fetuin-A, matrix *γ*-carboxylated glutamate protein, and *γ*-carboxylated glutamate-rich protein. Functionally, CPPs can be compared with the better-known lipoprotein particles, which scavenge and transport water-insoluble lipids to target tissues. Similarly, CPPs bind and sequester excess free calcium and phosphate ions, acting as a natural buffering system that helps regulate mineral homeostasis. By mediating the transport and clearance of these minerals, CPPs play a crucial role in preventing hypercalcemia, hyperphosphatemia, and pathologic mineral deposition in the extracellular space—most notably in the vasculature, contributing to arterial calcifications.^3^

The simplest form of these particles consists of a small calcium-phosphate cluster bound to a single fetuin-A molecule (58 kDa), known as calciprotein monomer (CPM), which seems to function as a physiologic transporter of excess calcium and phosphate. With a diameter of approximately 10 nm, CPMs are constitutively present in the bloodstream, particularly after high dietary phosphate intake, and are primarily cleared through glomerular filtration.^4^ However, when CPM clearance is impaired—such as in reduced glomerular filtration, leading to prolonged circulation—the interaction between fetuin-A, matrix *γ*-carboxylated glutamate protein, and glutamate-rich protein can promote the aggregation of multiple CPMs into larger, amorphous, proteinaceous spherical particles known as primary CPPs (CPP-I). Under certain stimuli, CPP-I undergo a transformation into needle-shaped crystalline secondary CPPs (CPP-II), containing calcium hydroxyapatite, through a process known as amorphous-to-crystalline transition. *In vitro* experiments have shown that this transition is decelerated by increased concentration of fetuin-A, albumin, pyrophosphate, and magnesium, while it is accelerated by higher temperature, pH, and concentration of calcium, phosphate, and lysozyme. Notably, reduced GFR, high phosphate levels, and deficiencies of pyrophosphate, fetuin-A, and albumin are all hallmarks of CKD. It is therefore not surprising that levels of CPP-I and CPP-II have been found higher in patients with CKD compared with healthy subjects.^5^

Owing to their larger size, CPP-I (50–100 nm) and CPP-II (120–200 nm) are not filtered by the glomerulus, nor cleared by dialysis, and are instead endocytosed by various cell types, including vascular endothelial and smooth muscle cells, adventitial fibroblasts, liver sinusoidal endothelial cells, and macrophages. Once internalized, CPPs may contribute to cell death and induce proinflammatory signaling and procalcifying effects. Experimental studies have demonstrated that CPPs play a role in endothelial dysfunction and vascular calcification. The molecular mechanisms underlying their internalization, intracellular signaling and pathologic effects are reviewed elsewhere.^6^ In clinical research, observational studies have shown that high CPP levels, measured using various methods, are associated with the development and progression of atherosclerosis, vascular calcification, and calciphylaxis. Moreover, the propensity of serum to form CPPs has been associated with the risk of cardiovascular events and mortality in patients with CKD^7^ and in kidney transplant recipients.^8^

Given this knowledge, an important question arises: what is the future of CPPs in nephrology? Could these particles become a valuable tool in clinical practice? To explore this, it is useful to consider the fundamental characteristics of an ideal biomarker. First, a biomarker must demonstrate analytical validity, meaning that it can be accurately and reliably measured in clinical laboratories, with high sensitivity, specificity, and reproducibility. Second, a biomarker must have clinical validity, signifying a clear and consistent association with disease processes and outcomes. Third, and perhaps most critically, a biomarker must possess clinical utility by improving risk stratification, guiding therapeutic decisions, or serving as a treatment target. Finally, practical considerations must be addressed, including cost-effectiveness, test availability, and potential ethical implications.

In this issue of *Kidney360*, the original research article by Tiong *et al.*^9^ provides valuable insights to this topic by evaluating the association between various simultaneous measurements of CPPs and the risks of mortality and disease progression in a cohort of 189 adult patients with CKD stages 3–4. In other words, they seek to clarify the differences among different CPP measurement methods and determine which of them show the strongest association with clinically relevant adverse outcome—an essential step in assessing the clinical validity of CPP assays. In particular, they use (*1*) a commercially available ELISA kit to measure “Fetuin-A CPPs,” which should include the total amount of CPP-I and CPP-II; (*2*) gel filtration techniques to measure “low-density CPPs (L-CPP),” which should correspond to CPMs, and “high-density CPPs,” which should include the total amount of CPP-I and CPP-II; and (*3*) flow cytometry to distinctly measure “CPP-I” and “CPP-II.”

One meaningful and reassuring finding is that all five CPP measures positively correlated with each other. However, each exhibited distinct associations with clinical outcomes. Notably, CPP-II was the most strongly associated with all-cause mortality. Unsurprisingly, high-density CPPs (which measure both CPP-I and CPP-II) also showed an association with mortality risk, likely driven by CPP-II. This finding confirms what experimental studies have suggested—that CPP-II may be the most harmful form of CPPs, capable of inducing cell death, inflammasome activation, endothelial dysfunction, and osteochondrogenic differentiation of vascular smooth muscle cells. These molecular mechanisms likely contribute to vascular stiffness and calcification, which in turn associate with the increased burden of cardiovascular disease and mortality, as well as accelerated CKD progression. In fact, in their analysis of secondary outcomes, the authors found that higher CPP-II levels were also associated with an increased risk of CKD progression. However, for this outcome, the strongest association was observed with L-CPP, representing CPMs. As the authors hypothesize, this observed effect may be mediated by an increase in fibroblast growth factor-23. Moreover, CPMs, which are filtered through the glomerulus, may aggregate into CPP-I and CPP-II in the presence of high phosphate concentrations in the tubular fluid, leading to tubular cell damage, interstitial inflammation, and fibrosis,^10^ thereby initiating a vicious cycle of nephron loss and disease progression.

This study offers valuable insights into the relative clinical significance of different CPP measurements, identifying CPP-II detected by flow cytometry as a potential risk factor for both all-cause mortality and CKD progression, while L-CPP detected by gel filtration emerged as a possible key risk factor for CKD progression. Furthermore, this work highlights the need for standardization of CPP measurements and their implementation in future research to assess their analytical validity in different cohorts of patients. However, some limitations must be acknowledged. The inclusion of patients with prevalent CKD raises the possibility of selection bias, and the relatively small sample size may limit the generalizability of the findings. Moreover, CPPs were measured at a single baseline time point, preventing an evaluation of their longitudinal trajectories and temporal associations with disease progression. Finally, the analyses did not account for potential confounding, mediation, or moderation by key mineral metabolism markers, such as calcium, phosphate, magnesium, PTH, and fibroblast growth factor-23, which could influence the observed associations. Thus, an important issue remains unsolved, which is whether measuring CPP-II or L-CPP levels would provide insights beyond current biomarkers such as serum calcium, phosphate, and PTH. If this was the case, further questions arise. Which levels of these new biomarkers should be considered safe? Is there a linear effect—the lower, the better—or is there a specific threshold above which risk for adverse outcome significantly increases? Which therapeutic strategies could modulate CPP formation or clearance? Would such interventions translate into better clinical outcomes?

As research continues to unfold, answering these questions will determine whether CPPs move from an intriguing biologic phenomenon to a routine component of nephrology practice.
